# Longitudinal Relationship Study of Depression and Self-Esteem in Postnatal Korean Women Using Autoregressive Cross-Lagged Modeling

**DOI:** 10.3390/ijerph17103743

**Published:** 2020-05-25

**Authors:** Jeong-Won Han, Da-Jung Kim

**Affiliations:** College of Nursing Science, Kyung Hee University, Seoul 02447, Korea; jung-321@hanmail.net

**Keywords:** depression, self-esteem, pregnancy, weight

## Abstract

Individuals with low self-esteem are vulnerable to depression. Depressed individuals process information related to themselves in a distorted way, thereby negatively affecting their self-esteem. The purpose of this study was to examine the relationship between depression and self-esteem in postnatal Korean women using longitudinal data and an autoregressive cross-lagged analysis. This study was conducted in postpartum women who had consistently participated in the Panel Study on Korean Children (PSKC) from Wave 1 through to Wave 8. The study results showed that depression and self-esteem in postnatal women had a significant positive correlation over time. Moreover, the longitudinal relationship between depression and self-esteem in postnatal women was affected by weight gain during pregnancy. This study overcomes the limitations of cross-sectional studies by using longitudinal data on the correlations between depression and self-esteem in postnatal women; the study findings may be used in developing weight control programs for pregnant and postnatal women.

## 1. Introduction

Approximately 30–70% of women who have given birth experience lowered self-esteem after childbirth due to various reasons. Lowered self-esteem causes an array of social and psychological roblems such as degraded marital relationships, depression, bulimia, and reduced childrearing competency [1]. Specifically, women who have positive self-esteem can easily overcome postpartum depression, however, those with a negative self-image experience worsened postpartum depression [2]. According to the cognitive vulnerability model of depression, which states that individuals with low self-esteem become vulnerable to depression when they experience failure or rejection by other people, an individual’s self-esteem is closely related to depression [3]. On the other hand, according to the wound model, depressed individuals process information related to themselves in a distorted way, thereby negatively affecting their self-esteem [4]. The cognitive vulnerability and wound models are not mutually exclusive, and it is possible to hypothesize by combining the two models that there is a reciprocal relationship between self-esteem and depression, whereby poor self-esteem in the past can have a significant effect on depression later, while high levels of depression in the past also has a significant effect on self-esteem later [4,5]. Low self-esteem in postnatal women is a crucial factor in the prevalence of postpartum depression [6]. Women with severe depression after childbirth are highly likely to have low self-esteem [7]; healthcare professionals should therefore be alert to the fact that self-esteem and depression in postnatal women need to be both monitored and managed [8].

Korean women gain an average of 13.6 kg during pregnancy, and maintain a weight gain of approximately 5.2 kg for a year after childbirth [9]. Moreover, about 28% of pregnant women with a normal body mass index (BMI) gain at least 20 kg, that is, 40% of their normal weight during pregnancy, which lasts for more than 6 months after childbirth [10]. A significant weight gain among pregnant women negatively affects their self-image, which may lead to neglecting their baby, suicidal thoughts, or even postpartum depression, which directly affecting their daily lives [11]. Depression in mothers is closely related to their own health as well as the growth and development of their child, and may influence the individual’s willingness to have children; factors that may cause postpartum depression should therefore be anticipated and managed in advance [12]. In this respect, healthcare professionals must recognize the fact that sudden weight gain during pregnancy is related to depression, and poor self-esteem in postpartum women; they should provide medical assistance for weight control during pregnancy and assess changes in mood and self-esteem levels in case of excessive weight gain [13].

In previous studies on weight gain during pregnancy, researchers at Victoria Hospital in the United Kingdom reported that weight gain during pregnancy causes various medical problems such as complications related to anesthesia, depression, and breastfeeding, thereby increasing medical costs [14]. According to research conducted at an OB-GYN clinic in East Massachusetts, USA, maintaining or losing weight after childbirth affects postpartum depression, which can hinder the return to one’s normal weight [15]. Furthermore, it has been reported that there is a causal relationship between BMI and postpartum depression among women in Norway, in which women with a higher BMI had 32% more severe depression than those with a lower BMI [16]. In another cohort study conducted in women in Denmark, it was discovered that weight gain before childbirth was related to postpartum depression, thus stressing the importance of weight control during pregnancy [17]. In studies targeting Korean women, weight gain due to pregnancy and childbirth was found to be harmful to women’s health, including postpartum recovery and postpartum depression [18]. Thus, it is clear that data from studies examining the relationship between sudden weight change during pregnancy and negative emotional states in women should be used to guide clinical interventions [19].

In recent years, there has been an increase in studies applying autoregressive cross-lagged modeling to clearly understand the reciprocal relationship between self-esteem and depression [5]. This can be interpreted as an attempt to overcome the limitations of a longitudinal study design for investigating this relationship, since emotional variables such as self-esteem or depression are known to continuously and irregularly change over time [20]. However, the number of studies investigating the reciprocal and longitudinal relationship between depression and self-esteem in postnatal Korean women has been limited, although this has a significant influence on the health of mothers and children. Our study hypothesis was that weight gain during pregnancy causes changes in the degree of depression and level of self-esteem; women were categorized into groups based on whether or not they had experienced weight gain. Longitudinal panel data on depression and self-esteem were analyzed using autoregressive cross-lagged modeling.

## 2. Materials and Methods

### 2.1. Research Design

This is a longitudinal study aimed to examine the relationship between depression and self-esteem in women who have given birth using secondary data analysis of data from the Panel Study on Korean Children (PSKC) conducted by the Korea Institute of Child Care and Education, Seoul, South Korea.

### 2.2. Research Subjects

The PSKC provided longitudinal data on babies born in 2008, their mothers, and the community environment. The PSKC included all households with babies born between April and July 2008 at sampled medical institutes whose annual number of deliveries exceeded 500 as of 2006. Households were excluded from the study if the mother could not communicate in Korean, the mother’s health after childbirth was extremely poor, the infant or mother had a serious illness, the infant was to be adopted, the infant was of a multiple birth, or if the mother was 18 years or younger. Moreover, sample households were excluded from tracking if the parents were separated from the child due to divorce or other separation, if the child had died, if the child was studying abroad, if tracking was not possible due to immigration, or if the household strongly desired to be excluded from sample tracking. The PSKC collected 2562 preliminary sample households for the study, of which 2150 were included in the final sample. The PSKC used a stratified multi-stage sampling method in which medical institutes where babies were born were selected in Stage 1, preliminary sample households were selected among the households with newborns in Stage 2, and final sample households were selected among the households that wished to participate in Stage 3. To ensure sample validity, the sample retention rate suggested by the PSKC was 96.7% after Wave 1, 88.6% after Wave 2, 83.8% after Wave 3, 81.6% after Wave 4, 79.2% after Wave 5, 77.3% after Wave 6, 75.3% after Wave 7, and 74.3% after Wave 8 of 2562 households. Our study targeted postpartum women who had consistently participated in the PSKC from Wave 1 through to Wave 8. In order to maintain the homogeneity of the subjects, 423 individuals who maintained the following status from Wave 1 to Wave 8 of the study were selected as the final subjects: (1) women of Korean nationality who were 19 years old or older, (2) women without disabilities, (3) women who were 145 cm or taller, (4) women who were legally married, (5) women who currently lived with their husbands, (6) women who did not have depression before childbirth, (7) women who had a planned pregnancy, (8) women who had their first child at the time of the study, (9) women with a pregnancy length of 37–41 weeks, (10) women whose children did not have disabilities, and (11) women whose children did not require treatment in an ICU at the time of birth. To conduct path analysis, the ideal size of the samples was required to be 200 or more; thus, in this study, 423 subjects was considered a sufficiently large cohort.

### 2.3. Measurements

#### 2.3.1. Depression

To measure depression, we used a questionnaire developed by Kessler (2002) [21], which was translated and adapted for the PSKC. The questionnaire comprised six questions (five-point scale), with higher scores indicating more severe depression. The degree of depression was determined based on the aggregate scores: 6–13 points, normal; 14–18 points, mild/moderate depression; 19–30 points, severe depression. Kessler’s questionnaire had a Cronbach’s alpha of 0.89, and PSKC Waves 1–8 had Cronbach’s alphas of 0.90–0.92.

#### 2.3.2. Self-Esteem

To measure self-esteem, we used Rosenberg’s (1965) [22] Self-Esteem Scale translated and adapted for the PSKC. It had a total of 10 questions (5-point scale), and a higher score indicated higher self-esteem. The reliability of the tool used in the preliminary study conducted among Korean women in 2007 had a Cronbach’s alpha of 0.82. The reliability of the PSKC’s wave 1 to wave 8 studies had Cronbach’s alphas of 0.86–0.89.

#### 2.3.3. Study Groups

During the preliminary panel study, we measured the subjects’ height, weight before pregnancy, and weight immediately before childbirth. Each item was measured and manually recorded by trained researchers who visited the OB-GYN clinic or postnatal care centers within one month of childbirth. BMI scores were calculated using the height and weight before pregnancy to categorize women into two groups: overweight and normal weight. Based on the Korean Ministry of Health and Welfare recommendations for total weight gain during pregnancy according to pre-pregnancy BMI, subjects who exceeded the recommended weight gain were categorized under the overweight group, and those who maintained the recommended weight gain were categorized under the normal group.

#### 2.3.4. Exogenous Variables

The exogenous variables in this study were parenting stress, household income, and employment status (this included students), which have been reported to affect depression in subjects over time based on Wave 8 PSKC data.

##### Parenting Stress

To measure parenting stress, the PSKC research team extracted a subfactor of Kim and Kang’s Parenting Stress Index (1997)—the pressure to fulfill the parental role; participants were asked to respond to a total of 11 questions drafted based on the preliminary 2007 study on a 5-point scale, with a higher score indicating a higher level of parenting stress. Kim and Kang’s Parenting Stress Index (1997) had a Cronbach’s alpha of 0.86, and Cronbach’s alphas of 0.82–0.89 in PSKC Waves 1–8.

##### Household Income

Household income in the PSKC included all earned income, financial assets, business profits, rental income, transferred income, and any income from other sources. Income referred to the total income earned by all household members; net income did not include income tax, residence tax, property tax, interest income tax, national pension, or national health insurance. The only question used to collect data on household income was “What was the average monthly household income for the past year?”

##### Employment Status

Employment status (including being a student) was determined as in the PSKC, where individuals who were on maternity leave or a leave of absence due to sickness or accidents, working part-time, or working more than 30 h a week at a place operated by her family without receiving wages were all considered employed. Individuals who were on a leave of absence from school were considered as studying. All other subjects were categorized as unemployed or not attending school.

### 2.4. Data Collection and Analysis 

The data used in this study were obtained by first submitting a research protocol through the PSKC website (http://panel.kicce.re.kr) operated by the Korea Institute of Child Care and Education; approval was obtained after deliberation by the committee. This study was approved by the Institutional Review Board of Kyung-Hee University, Seoul, South Korea (KHSIRB-19-206 (EA)). SPSS WIN 24.0 (SPSS Korea Data Solution, Inc. Seoul, South Korea) and AMOS 18.0 (SPSS Korea Data Solution, Inc., Seoul, South Korea) were used for entering and analyzing the data. General characteristics of the subjects were analyzed using absolute numbers, percentage, mean, and standard deviation. The level of depression and self-esteem of the subjects by measurement point was analyzed using mean and standard deviation, while the relationship between the test values was analyzed using Pearson’s correlation coefficient. The reliability of the questionnaires was measured using Cronbach’s alpha coefficient. The causal relationship between depression and self-esteem in postnatal women over time were examined using longitudinal data and autoregressive cross-lagged analysis. Multiple aspects of the relationship between the two variables were explored to deduce an apparent relationship. The autoregressive cross-lagged model comprises an autoregressive component and a cross-lagged component. The cross-lagged component indicates the interaction between two variables over an extended period of time. In this study, the relationship between depression and self-esteem in postnatal women was investigated using autoregressive cross-lagged analysis in seven steps. Each model had an intrinsic relationship with the other models to which difference tests could be applied; the model’s normed fix index (NFI), comparative fit index (CFI), Tucker-Lewis Index (TLI), and root mean squared error of approximation (RMSEA) goodness-of-fit were estimated as these are sensitive to the sample size. The models are explained in greater detail as follows: Model 1: Basic model; Model 2: Covariance added to the structural error of Model 1; Model 3: Identification constraint added to the autoregressive coefficient of depression in Model 2; Model 4: Identification constraint added to the autoregressive coefficient of self-regression in Model 3; Model 5: Identification constraint added to the cross-lagged coefficient of depression with respect to self-esteem in Model 4; Model 6: Identification constraint added to the cross-lagged coefficient of self-esteem with respect to depression in Model 5; Model 7: Identification constraint added to the error covariance of depression and self-esteem in Model 6. The maximum likelihood method was used to verify the final model’s goodness-of-fit. For the model’s goodness-of-fit test, the goodness-of-fit index Chi-square (χ^2^), degree of freedom (df), RMSEA, standardized root mean square residual (SRMR), CFI, NFI, and TLI were used. A test of the structural model’s invariance across the groups was conducted to verify if the causal relationship between depression and self-esteem in the subjects was affected by weight gain during pregnancy, in which the critical ratios for difference in the free model and constraint model were compared. 

## 3. Results

### 3.1. General Characteristics of the Subjects

The average age of the subjects was 30.59 ± 3.6 years, and the average duration of marriage was 30.30±23.8 months. With regard to the education level of the subjects, 101 subjects (23.9%) were high school graduates or less, 130 (30.7%) had a 2-year college degree, and 192 (45.4%) had a bachelor’s degree or higher; 192 subjects (45.4%) had no religion, whereas 231 (54.6%) were religious. With respect to reproduction techniques used during childbirth, 396 subjects (93.6%) conceived naturally, and 27 (6.4%) received assistance; 228 subjects (53.9%) had normal spontaneous delivery, 138 (32.6%) had unplanned emergency C-sections, and 57 (13.5%) had planned C-sections. Prior to pregnancy, 70 subjects (16.5%) had a BMI of less than 18.5 kg/m^2^, 313 (74.0%) had a BMI of 18.5–24.9, and 40 (9.5%) had a BMI of 25 or higher. Based on the recommendations for total weight gain during pregnancy based on pre-pregnancy BMI, 98 subjects (23.2%) were overweight and 325 (76.8%) experienced normal weight gain (Table 1).

### 3.2. Normality Test of Measurement Variables

All variables had an absolute skewness value of less than 3, as well as an absolute kurtosis value of less than 10 every year, thereby passing the normality test (Table 2). Mean values for depression gradually increased and then decreased, whereas those of self-esteem slowly decreased and then increased over the 8-year study period. 

### 3.3. Correlation between the Measurement Variables

According to the analysis of the correlation between the measurement variables, depression and self-esteem had a statistically significant correlation throughout the study period from Waves 1 to 8 (Table 3). The subjects’ depression and self-esteem were found to have a significant correlation with exogenous variables (parenting stress; r = −0.29−0.66 and household income; r = −0.14−0.21).

### 3.4. Relationship between the Measurement Variables

**Figure 1:**
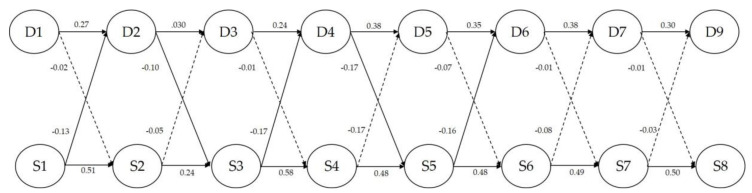
The longitudinal relationship between depression and self-esteem. D1 = Depression 1st, D2 = Depression 2nd, D3 = Depression 3rd, D4 = Depression 4th, D5 = Depression 6th, D7 = Depression 7th, D8 = Depression 8th, S1 = Self-esteem 1st, S2 = Self-esteem 2nd, S3 = Self-esteem 3rd, S4 = Self-esteem 4th, S5 = Self-esteem 6th, S7 = Self-esteem 7th, S8 = Self-esteem 8th, ―> Significantly, ∙∙∙∙∙> Non-Significantly

In this study, the correlation between depression and self-esteem in postnatal women was estimated using autoregressive cross-lagged analysis in seven steps. As the goodness-of-fit index remained adequate under the identification constraint, we assumed that identity had been established. As the goodness-of-fit indices of Models 6 and Model 7 did not have a significant difference; Model 7 was selected as the final model. The goodness-of-fit of Model 7 was χ^2^ = 408.34, df = 114, RMSEA = 0.002, SRMR = 0.002, CFI = 0.90, NFI = 0.87, and TLI = 0.89; the structural coefficient is given in Table 4. Depression had a statistically significant positive correlation with time (respectively β = 0.25, β = 0.27, β = 0.27, β = 0.36, β = 0.35, β = 0.38, β = 0.30, *p* < 0.001), as did self-esteem (respectively β = 0.50, β = 0.22, β = 0.51, β = 0.44, β = 0.47, β = 0.49, β = 0.50, *p* < 0.001). In the analysis of the cross-lagged effects between depression and self-esteem, depression in the second wave had a statistically significant effect on self-esteem in the third wave (β = −0.17, *p* = 0.025), depression in the fourth wave on self-esteem in the fifth wave (β = −0.26, *p* = 0.049), self-esteem in the first wave on depression in the second wave (β = −0.17, *p* = 0.007), self-esteem in the third wave on depression in the fourth wave (β = −0.25, *p* < 0.001), and self-esteem in the fifth wave on depression in the sixth wave (β = −0.17, *p* = 0.040) (Table 4) (Figure 1).

### 3.5. Difference in Weight Gain during Pregnancy between Groups

In this study, subjects who exceeded the recommended weight gain were categorized in the overweight group, while those who experienced the recommended weight gain were categorized in the normal group based on the Korean Ministry of Health and Welfare’s recommendations for total weight gain during pregnancy as a proportion of pre-pregnancy BMI. The critical ratio for the difference in the free model and the constraint model of the path in the research model was calculated to examine the statistically significant difference between the path coefficients of the two groups. The results showed that the path from depression in the seventh wave to depression in the eighth wave (critical ratio for difference = −3.204, *p* < 0.001), the path from self-esteem in the sixth wave to self-esteem in the seventh wave (critical ratio for difference = −3.092, *p* < 0.001), and the path from self-esteem in the seventh wave to self-esteem in the eighth wave (critical ratio for difference = −2.608, *p* < 0.001) were statistically significantly different. In the normal weight group, depression in the seventh wave did not have a significant effect on depression in the eighth wave (β = 0.08, *p* = 0.319), whereas in the overweight group, depression in the seventh wave had a significant effect on depression in the eighth wave (β = 0.39, *p* < 0.001). 

Furthermore, in the normal weight group, the standardized coefficient of self-esteem in the sixth wave and self-esteem in the seventh wave was β = 0.67 (*p* < 0.001), and that of self-esteem in the seventh wave and self-esteem in the eighth wave was β = 0.65 (*p* < 0.001), whereas in the overweight group, the standardized coefficient of self-esteem in the sixth wave and self-esteem in the seventh wave was β = 0.39 (*p* < 0.001), and that of self-esteem in the seventh wave and self-esteem in the eighth wave was β = 0.42 (*p* < 0.001), thus exhibiting a difference in standardized coefficients between the two groups (Table 5).

## 4. Discussion

The purpose of this study was to examine the causal relationship between depression and self-esteem in postnatal women in Korea over time using longitudinal data from the PSKC. First, we found that both depression and self-esteem in postnatal women were positively correlated with changes in time. These results are similar to the findings of a previous study conducted in the United Kingdom [14], which found that women tend to have a negative self-image after childbirth that may cause depression that lasts for a certain period of time. Previous research on 240 women in the United States from pregnancy to 6 weeks after childbirth [15] also found that depression in postnatal women becomes worse due to concerns about not being able to return to pre-pregnancy weights. As estrogens are hormones that control the generation and breakdown of fat cells, women who experience hormonal changes during pregnancy and childbirth also undergo changes in body shape due to increased body fat [23]. These physical changes also affect women’s emotional state, particularly depression, which lasts over a long period of time or occurs repeatedly [24]. Therefore, healthcare professionals should evaluate and intervene in case of disturbed emotional states in pregnant women such as depression from early pregnancy; in particular, they should investigate whether these psychological changes are related to changes in body weight. The results of this study are similar to those of a study that followed up on 100 Moroccan women from the first 3 months of pregnancy to 9 months after childbirth [25], in which self-esteem in these women changed over time. Such results show that negative physical changes in women can reduce self-esteem and self-confidence. Women tend to experience heightened emotional anxiety and lowered self-esteem in case of body image discrepancy; heightened anxiety and lowered self-esteem ultimately cause coping mechanisms to become ineffective after childbirth, thereby hindering child-rearing activities and the development of the child in the long run [26]. Healthcare professionals should therefore accurately assess the emotional state of pregnant women even during pregnancy and take appropriate steps to offset the heightened risk of depression or lowered self-esteem, by studying how these emotional states are related to negative body image due to weight gain.

Second, according to the cross-lagged effects between depression and self-esteem, depression in the subjects in the second wave had a significant effect on self-esteem in the third wave, depression in the fourth wave on self-esteem in the fifth wave, self-esteem in the first wave on depression in the second wave, self-esteem in the third wave on depression in the fourth wave, and self-esteem in the fifth wave on depression in the sixth wave. These results are similar to the findings of a previous study conducted on postnatal women in Korea [27], which reported that self-esteem in new mothers was negatively correlated with depression, and of another longitudinal study [28,29] investigating postnatal women in Melbourne (Australia) for five years, which reported that as depression in women becomes more severe, their self-esteem is lowered. That is, the level of self-esteem or depression in the past have mutually reciprocal effects on levels at a later point in time [5,30]. Lowered self-esteem in women worsens depression, and more severe depression in return causes negative self-perception, thus lowering self-esteem even further. The post-childbirth period is especially difficult for women. Women need time to recover physically and adjust emotionally and socially as this is when most experience confusion from having to fulfill a new role and bear new responsibilities, and this can lead to a developmental crisis [31,32]; if women are unable to adjust well during this period, they are highly likely to develop depression due to psychological problems such as lowered self-esteem. This study showed that the interaction between depression and self-esteem in postnatal women had significant effects from Waves 1 to 6, which is when their child reached the age of 5, and this result is consistent with that of a study by Freidman and Resnick [28], which reported that depression in mothers was aggravated as the child grows through infancy. It is also consistent with a study by Woolhouse et al. [29] that followed up women in Australia for four years; the authors reported that more mothers suffered from depression four years after childbirth than they did one year after childbirth. This is related to the fact that postnatal women suffer from various physical problems after childbirth [33,34] but do not have sufficient time for self-care because they are constantly involved in parenting [35]. Children between the ages of one and five grow rapidly and satisfy their desires mostly through their mothers, which may explain why mothers undergo psychological changes from the parental burden of childrearing [36]. The risk of postpartum depression in Korean culture is devalued, even though the frequency of Korean women experiencing postpartum depression is similar to that of Western women. Also, in Korea, even if a woman has postpartum depression, the chances of her seeking professional help are lower than in Western culture [37]. Healthcare professionals should therefore develop and implement psychological therapy programs for women during pregnancy up to the period after childbirth in order to improve the mental and physical health of women and children. In addition, long-term assistance for postnatal women should be provided at the community level.

Third, the study results showed that in the normal weight group, the degree of depression in the seventh wave did not have a significant effect on depression in the eighth wave, whereas in the overweight group, the degree of depression in the seventh wave did have a significant effect on depression in the eighth wave; the standardized coefficient of self-esteem in Wave 6 on self-esteem in Wave 7 and self-esteem in Wave 7 on self-esteem in Wave 8 was higher in the normal weight group than in the overweight group. This result corresponds with the findings of a previous study in postnatal women in Norway [16], which reported that women with a higher BMI were more likely to have postpartum depression, and the results of another study in postnatal women in Morocco [25], which reported that women’s self-esteem changed over time. Many women are worried about not being able to return to their prepartum normal weight; in particular, women who become overweight during pregnancy are at a higher risk of developing complications during pregnancy from the increased weight as well as long-term depression than women with normal weight [38]. Moreover, there is still pressure in Korean society to be slim, especially on women; thus, being overweight or obese has a negative effect on body image and lowers self-confidence. Negative self-perception, which is linked with depression, may worsen in this context [39]. Ultimately, depression and self-esteem in postnatal women are important factors affecting the growth and development of children, thus requiring active intervention by healthcare professionals. Our study results indicate that depression and self-esteem in postnatal women are related to excessive weight gain during pregnancy; since depression and self-esteem do not improve or change within a short period of time, support from the medical community and the community at large should be provided to pregnant and postnatal women. Moreover, weight control during pregnancy should be managed by the individual herself; however, this also requires the establishment of various supportive systems for depression and self-esteem in postnatal women. As depression in overweight women lasted until Wave 8 in this study, depression in mothers may affect their child’s social life, since children start school at age seven in Korea. A successful social life in one’s early years leads to a successful social life in one’s adult years; thus, practical and active measures should be taken to ensure the psychological health of pregnant and postnatal women.

This means that the mental health of women is not affected by the biological changes that occur during pregnancy and childbirth alone, but is of a different order from men in terms of social, economic and cultural factors, and that mental health conditions in Korea have a negative effect on women. Women are more likely to experience stress if they are unable to return to their pre-childbirth body shape, and those who become overweight during pregnancy are more likely to develop complications due to increased weight, and suffer from depression for a longer period of time than those who are able to maintain normal weight [38]. Also, because slimness is still considered culturally desirable, overweight and obesity can have a negative effect on relative emotional deprivation and physical satisfaction, which can further reduce self-esteem and increase negative self-awareness and thereby aggravate depression [39]. Especially, the continuous appearance of depression in overweight women during pregnancy is limited to the problem of human illness by returning to the problem of hormone change in women by experts in the field of mental health for depression in childbirth women, this is because women’s individualization is the main goal of adapting to the environment, and they overlook the social relationship between production and reproduction that determines women’s mental health [40]. According to the Seoul Citizens’ Perception Survey on mental health, women of received greater help and counseling from others in relation to their mental problems than men, but the nature of the counseling received was mostly informal and was provided by friends, acquaintances, family members or relatives [41]. Although this suggests that women enjoy stronger personal networks than men, this is still not adequate for resolving mental health problems in women.

## 5. Conclusions

This study examined the longitudinal relationship between depression and self-esteem in postnatal women in Korea using data from the PSKC. The study results showed that depression and self-esteem in postnatal women had a significant positive correlation over time. Moreover, the longitudinal relationship between depression and self-esteem in postnatal women was affected by weight gain during pregnancy. This study overcomes the limitations of cross-sectional studies as it is a longitudinal study of the correlation between depression and self-esteem in postnatal women; our findings could be used to develop weight control intervention programs for pregnant and postnatal women based on the amount of weight gain (normal or excessive) during pregnancy. However, a limitation of this study is that we could not simultaneously examine changes in the level of depression and self-esteem in postnatal women and their relationship with the changes in postpartum body weight, as only previously collected panel study data were used. We suggest future research into the relationship between depression and self-esteem in postnatal Korean women should also focus on other aspects of women’s mental health such as the degree of couple connectedness, nature of the marital relationship, whether or not the mother is sleep deprived, and the quality of the partner’s support and the mother’s social support network, as these could also affect the emotional state of the mother. 

## Figures and Tables

**Table 1:** General characteristics (*N* = 423).

Variables		*n*	%
Age, years	<30	181	42.9
(Mean ± SD = 30.59 ± 3.60)	30–35	197	46.6
	<35	45	10.5
Duration of marriage, months	≤12	53	12.6
(Mean ± SD = 30.30 ± 23.80)	13–24	179	42.4
	25–36	100	23.7
	≤37	91	21.3
Education level	High school graduate or less	101	23.9
	2-year college degree	130	30.7
	Bachelor’s degree or higher	192	45.4
Religion	No	192	45.4
	Yes	231	54.6
of Reproduction assistance techniques used	Naturally conceived	396	93.6
	Received assistance	27	6.4
Delivery type	Normal spontaneous delivery	228	53.9
	Unplanned emergency C-sections	138	32.6
	Planned emergency C-sections	57	13.5
BMI (before pregnancy), kg/m^2^	<18.5	70	16.5
	18.5–24.9	313	74.0
	<25.0	40	9.5
Total weight gain during pregnancy, kg	Overweight	98	23.2
	Maintained normal weight gain	325	76.8

SD = standard deviation.

**Table 2:** Descriptive statistics.

Variables	Mean	SD	Skewness	SE	Kurtosis	SE
Depression wave 1	1.78	0.66	0.93	0.12	1.34	0.24
Depression wave 2	1.75	0.63	0.65	0.12	0.30	0.24
Depression wave 3	1.82	0.58	0.65	0.12	1.30	0.24
Depression wave 4	1.86	0.66	0.70	0.12	0.34	0.24
Depression wave 5	1.88	0.64	0.61	0.12	0.19	0.24
Depression wave 6	1.86	0.64	0.67	0.12	0.47	0.24
Depression wave 7	1.82	0.66	0.77	0.12	0.85	0.24
Depression wave 8	1.72	0.63	1.21	0.12	2.14	0.24
Self-esteem wave 1	3.77	0.47	−0.21	0.12	0.31	0.24
Self-esteem wave 2	3.74	0.48	−0.45	0.12	2.14	0.24
Self-esteem wave 3	3.71	0.45	0.10	0.12	0.66	0.24
Self-esteem wave 4	3.71	0.55	−0.02	0.12	-0.18	0.24
Self-esteem wave 5	3.77	0.52	−0.32	0.12	0.10	0.24
Self-esteem wave 6	3.81	0.52	−0.18	0.12	0.18	0.24
Self-esteem wave 7	3.83	0.53	−0.23	0.12	0.03	0.24
Self-esteem wave 8	3.86	0.52	−0.36	0.12	0.34	0.24
Parenting stress wave 1	2.52	0.58	0.05	0.11	0.29	0.24
Parenting stress wave 2	2.48	0.58	0.10	0.11	0.26	0.24
Parenting stress wave 3	2.63	0.54	0.10	0.11	0.66	0.24
Parenting stress wave 4	2.64	0.60	0.03	0.11	0.18	0.24
Parenting stress wave 5	2.63	0.61	0.18	0.11	0.06	0.24
Parenting stress wave 6	2.58	0.58	-0.08	0.11	0.02	0.24
Parenting stress wave 7	2.51	0.57	0.14	0.11	0.06	0.24
Parenting stress wave 8	2.33	0.61	0.17	0.11	0.12	0.24
Household income wave 1	2622.32	1106.71	1.32	0.12	2.77	0.24
Household income wave 2	32596.15	1052.17	1.06	0.12	2.45	0.24
Household income wave 3	3108.26	1505.09	1.89	0.12	2.94	0.24
Household income wave 4	3243.82	1179.80	1.60	0.12	2.40	0.24
Household income wave 5	3149.34	1072.62	1.53	0.12	0.24	0.24
Household income wave 6	2624.19	1222.27	1.65	0.12	2.32	0.24
Household income wave 7	2900.30	1563.37	2.05	0.12	2.49	0.24
Household income wave 8	2828.65	1641.94	1.83	0.12	2.95	0.24

SD = standard deviation, SE = standard error. Household income: unit = US dollar.

**Table 3:** Correlation between depression and self-esteem.

Variables	X1	X2	X3	X4	X5	X6	X7	X8	X9	X10	X11	X12	X13	X14	X15	X16
X1	1															
X2	0.46 *	1														
X3	0.42 *	0.40 *	1													
X4	0.46 *	0.41 *	0.42 *	1												
X5	0.46 *	0.45 *	0.40 *	0.49 *	1											
X6	0.41 *	0.37 *	0.41 *	0.40 *	0.53 *	1										
X7	0.48 *	0.42 *	0.40 *	0.42 *	0.50 *	0.56 *	1									
X8	0.39 *	0.33 *	0.33 *	0.37 *	0.40 *	0.39 *	0.50 *	1								
X9	−0.53 *	−0.40 *	−0.23 *	−0.34 *	−0.32 *	−0.28 *	−0.43 *	−0.29 *	1							
X10	−0.34 *	−0.45 *	−0.21 *	−0.23 *	−0.27 *	−0.25 *	−0.31 *	−0.26 *	0.58 *	1						
X11	−0.40 *	−0.34 *	−0.53 *	−0.39 *	−0.36 *	−0.36 *	−0.41 *	−0.32 *	0.51 *	0.41 *	1					
X12	−0.40 *	−0.34 *	−0.32 *	−0.50 *	−0.31 *	−0.29 *	−0.34 *	−0.32 *	0.56 *	0.50 *	0.63 *	1				
X13	−0.38 *	−0.35 *	−0.29 *	−0.36 *	−0.56 *	−0.36 *	−0.43 *	−0.28 *	0.50 *	0.49 *	0.54 *	0.61 *	1			
X14	−0.31 *	−0.31 *	−0.25 *	−0.27 *	−0.37 *	−0.48 *	−0.45 *	−0.32 *	0.46 *	0.47 *	0.45 *	0.56 *	0.63 *	1		
X15	−0.28 *	−0.26 *	−0.30 *	−0.29 *	−0.37 *	−0.36 *	−0.53 *	−0.33 *	0.48 *	0.39 *	0.49 *	0.52 *	0.58 *	0.64 *	1	
X16	–0.29 *	–0.28 *	–0.33 *	–0.29 *	–0.35 *	–0.32 *	–0.43 *	–0.48 *	0.43 *	0.44 *	0.53 *	0.59 *	0.55 *	0.61 *	0.66 *	1

X1 = Depression 1st, X2 = Depression 2nd, X3 = Depression 3rd, X4 = Depression 4th, X5 = Depression 5th, X6 = Depression 6th, X7 = Depression 7th, X8 = Depression 8th, X9 = Self-esteem 1st, X10 = Self-esteem 2nd, X11 = Self-esteem 3rd, X12 = Self-esteem 4th, X13 = Self-esteem 5th, X14 = Self-esteem 6th, X15 = Self-esteem 7th, X16 = Self-esteem 8th, * *p* < 0.001.

**Table 4:** Model fitness comparison.

Model	χ^2^	df	NFI	CFI	TLI	RMSEA
Model 1	812.17	91	0.77	0.78	0.70	0.07
Model 2	308.23	84	0.90	0.92	0.84	0.03
Model 3	316.94	90	0.90	0.92	0.86	0.02
Model 4	372.79	98	0.89	0.91	0.86	0.02
Model 5	377.28	102	0.89	0.91	0.87	0.02
Model 6	391.20	108	0.88	0.91	0.88	0.02
Model 7	408.34	114	0.87	0.90	0.89	0.02

df = degree of freedom, NFI = Normed Fit Index, CFI = Comparative Fit Index, TFI = Turker–Lewis Index, RMSEA = root mean square error of approximation.

**Table 5:** Parameter estimation of the relationship between depression and self-esteem.

Pathway	*B*	β	CR	*p*	Critical Ratio for Difference
Depression 1st -> Depression 2nd	0.25	0.27	5.44	<0.001	−0.402
Depression 2nd -> Depression 3rd	0.27	0.30	6.28	<0.001	−0.889
Depression 3rd -> Depression 4th	0.27	0.24	5.13	<0.001	−0.434
Depression 4th -> Depression 5th	0.36	0.38	8.47	<0.001	0.273
Depression 5th -> Depression 6th	0.35	0.35	7.89	<0.001	0.104
Depression 6th -> Depression 7th	0.38	0.38	8.94	<0.001	−0.304
Depression 7th -> Depression 8th	0.30	0.30	7.05	<0.001	−3.204 *
Self-esteem 1st -> Self-esteem 2nd	0.50	0.51	10.87	<0.001	0.345
Self-esteem 2nd -> Self-esteem 3rd	0.22	0.24	5.15	<0.001	1.662
Self-esteem 3rd -> Self-esteem 4th	0.51	0.58	14.09	<0.001	0.432
Self-esteem 4th -> Self-esteem 5th	0.44	0.48	11.32	<0.001	−1.343
Self-esteem 5th -> Self-esteem 6th	0.47	0.48	11.11	<0.001	−0.460
Self-esteem 6th -> Self-esteem 7th	0.49	0.49	11.64	<0.001	−3.092 *
Self-esteem 7th -> Self-esteem 8th	0.50	0.50	12.67	<0.001	−2.608 *
Depression 1st -> Self-esteem 2nd	−0.01	−0.02	0.39	0.699	0.050
Depression 2nd -> Self-esteem 3rd	−0.17	−0.10	−2.25	0.025	0.381
Depression 3rd -> Self-esteem 4th	−0.01	−0.01	−0.24	0.972	0.496
Depression 4th -> Self-esteem 5th	−0.26	−0.17	−1.65	0.049	−0.852
Depression 5th -> Self-esteem 6th	−0.05	−0.07	−1.61	0.108	1.148
Depression 6th -> Self-esteem 7th	−0.01	−0.01	−0.24	0.813	−0.453
Depression 7th -> Self-esteem 8th	−0.01	−0.01	−0.29	0.771	−1.608
Self-esteem 1st -> Depression 2nd	−0.17	−0.13	−2.69	0.007	−1.794
Self-esteem 2nd -> Depression 3rd	−0.06	−0.05	−0.97	0.332	−1.323
Self-esteem 3rd -> Depression 4th	−0.25	−0.17	−3.60	<0.001	−0.626
Self-esteem 4th -> Depression 5th	−0.06	−0.17	−1.09	0.275	0.808
Self-esteem 5th -> Depression 6th	−0.17	−0.16	−1.71	0.040	−0.343
Self-esteem 6th -> Depression 7th	−0.09	−0.08	−1.75	0.081	−0.409
Self-esteem 7th -> Depression 8th	–0.04	–0.03	–0.79	0.429	–1.794

Depression 1st, 2nd, 3rd, etc. = degree of depression in Wave 1, 2, 3, etc., self-esteem 1st, 2nd, 3rd, etc. = level of self-esteem in Wave 1, 2, 3, etc., * There was statistically significant difference between the path coefficients of the two groups.
